# VISTA: bridging gaps in cancer immunotherapy

**DOI:** 10.1007/s12672-026-04407-4

**Published:** 2026-02-10

**Authors:** Aida Khademolhosseini, Mina Roshan Zamir, Ali Moazzeni, Zahra Mansourabadi, Elahe Safari

**Affiliations:** 1 Shiraz Institute for Cancer ResearchSchool of Medicine, Shiraz University of Medical Sciences, Shiraz, Iran; 2 Department of HematologyFaculty of Allied Medicine, Bushehr University of Medical Sciences, Bushehr, Iran; 3 Department of ImmunologySchool of Medicine, Iran University of Medical Sciences, Tehran, Iran; 4 Breast Health & Cancer Research Center, Iran University of Medical Sciences, Tehran, Iran

**Keywords:** VISTA, Cancer, Immune checkpoint, Immunotherapy, Tumor microenvironment

## Abstract

Tumor microenvironment (TME) is complicated by the interaction of different cells of immune system, stromal components, and tumor-associated elements. Immune cells largely influence tumor progression by the means of various activating and inhibitory mechanisms including, immune checkpoint molecules. These molecules have been targeted for treating different types of cancers. For instance, blocking antibodies against CTLA-4, PD-1, or PD-L1 have elicited durable clinical responses and remarkable efficacy. These antibodies have also led to long-term remissions in a subset of patients, especially when used in combination therapies. V-domain immunoglobulin suppressor of T cell activation (VISTA) as a negative regulator of the immune system is expressed on multiple immune cell subsets including, myeloid-derived suppressor cells (MDSCs), macrophages, and lymphocytes. VISTA exerts regulatory effects and modulates T cell function and has shown prognostic significance in different cancers, leading to an increased attention regarding its suppressive role in the context of cancer. In this review, we will summarize the VISTA structure, ligands, role in the TME, and expression on immune cells. Furthermore, the significance of VISTA expression in the prognosis of cancer and its role in cancer immunotherapy, tumor resistance and ongoing clinical trials will be discussed.

## Background

Cancer is the major health challenge and concern of the world, and currently, immunotherapy tends to be the most promising tumor therapy [1, 2]. National Center for Health Statistics in United States reported that, 1,958,310 new cancer cases and 609,820 cancer deaths were projected to occur in 2023 [3]. A major breakthrough in cancer immunotherapy is immune checkpoint inhibitors (ICIs), which take advantage of immune cells to fight against tumor cells [4]. Immunological checkpoints are groups of inhibitory or stimulatory pathways that affect immune system responses [5]. Several antibodies targeting inhibitory receptors, including cytotoxic T-lymphocyte-associated protein 4 (CTLA-4), programmed cell death protein 1 (PD-1), and programmed cell death-ligand 1 (PD-L1), as negative regulators of T cell immune function have revolutionized cancer immunotherapy [6]. Tumor cells exploit these inhibitory molecules in order to induce tumor tolerance and T cell exhaustion [7] thus, inhibition of these targets results in enhanced activation of the immune system and has been approved for the treatment of different cancers including non-small cell lung cancer (NSCLC) [8], melanoma [9], renal and bladder cancers [10]. Moreover, several other therapies targeting these molecules are in advanced stages of development [11, 12]. Despite the advantages, only a subset of patients (20–40%), benefit from these therapies, highlighting the growing need to develop new targets [13].

V-domain immunoglobulin suppressor of T cell activation (VISTA) as a negative immune checkpoint protein is highly expressed on tumor-infiltrating T cells, CD11b^+^ myeloid cells including, CD14^+^ monocytes, neutrophils, macrophages and myeloid CD11c^+^ dendritic cells (DCs), CD68^+^ macrophages and myeloid-derived suppressor cells (MDSCs) [14–16]. It is also expressed on naïve CD4^+^ and CD8^+^ T cells, CD4^+^/Foxp3^+^ Tregs, and CD56^low^NK cells to a lower extent [17]. The unique constitutive pattern expression of VISTA in steady states highlights its important role in immune system regulation and makes it striking in cancer immunotherapy [18]. VISTA is highly conserved in different species and has particularly structural homology to PD-1; however it possesses some unusual structural features [19, 20]. Studies on VISTA knockout mice have demonstrated its inhibitory role both as a ligand on antigen-presenting cells (APCs) and as a receptor on T cells [21], following its binding to different receptors in various ranges of pH [22]. This protein has emerged as a potential target in cancer immunotherapy, especially in combinational therapy, both in mouse models and pre-clinical studies [23–26]. Currently, small molecules or monoclonal antibodies (mAbs) directed against VISTA have been used in phase I and II of clinical trials and have revealed acceptable tolerability profiles and clinical activity [19]. Moreover, VISTA has represented prognostic implication value as a biomarker in different solid tumors [27–29]. Meta-analysis has revealed that high expression of VISTA is associated with favorable overall survival. In addition, increased expression of VISTA is significantly correlated with higher numbers of CD8^+^ tumor-infiltrating lymphocytes [30, 31]. Therefore, reviewing VISTA’s structure, function, role in tumor suppression, prognostic value, and clinical implications would draw attention to its outstanding feature in cancer immunotherapy.

## VISTA structure and function

**Fig. 1:**
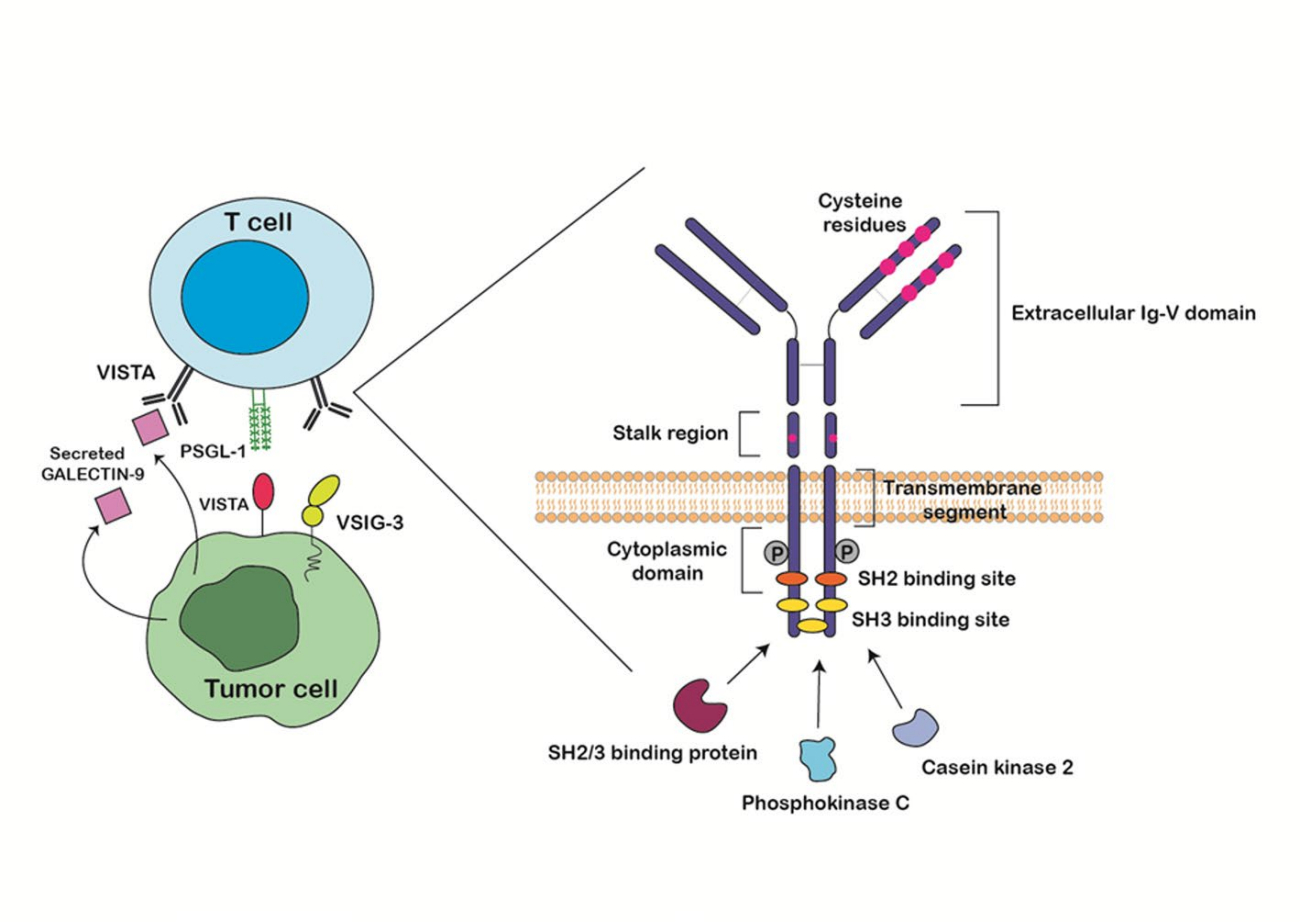
VISTA structure and its downstream signaling. VISTA: V-domain Ig suppressor of T cell activation. VSIG3: V-Set and Immunoglobulin domain containing 3. PSGL-1: P-selectin glycoprotein ligand-1

VISTA, also called D1Α, c10orf54, B7-H5, stress-induced secreted protein-1 (SISP1), differentiation of embryonic stem cells 1 (Dies1) and programmed death protein-1 homolog (PD-1 H) is a type I immunoglobulin membrane protein [32]. The protein is encoded by the VSIR gene on chromosome 10 (10q22.1) which is located inside the intron of the CDH23 gene with a ∼35 to 45 kDa molecular mass and is 279 amino acids long [33, 34]. Human VISTA contains a signal peptide (~ 32 aa), a single extracellular Ig-V domain (~ 130 aa) in the extracellular region with five cysteine residues, followed by a stalk region (~ 30 aa) with an invariant cysteine, a transmembrane domain (~ 21 aa), and a long cytoplasmic domain (~ 96 aa), respectively (Fig. 1) [35, 36]. Extracellular Ig-V domain is hyper-glycosylated, containing three disulfide bonds where all cysteine residues are located [37]. Besides, the extracellular domain (ECD) has a significant number of histidine residues which are mostly conserved and located in the complementarity-determining region -proximal half of the molecular surface. These surface-exposed histidine clusters are required for T cell suppression [38]. VISTA multimers preferably bind to leukocytes at acidic pH (pH < 6.5), where the histidine residues are positively charged, and not in the physiological one (pH = 7.4) [22]. Recent studies also demonstrated that extracellular Ig-V-like domain of VISTA is larger than expected (149 aa), probably due to an additional “H” β-strand, “clamping” disulfide and a long C-C′ loop which may be important for PD-1 H regulation and function [38]. Transforming growth factor beta type 1 (TGF-β)-Smad3 signaling pathway is primarily responsible for controlling VISTA expression. Smad3 could directly bind to VISTA promoter gene and siRNA-mediated Smad3 knock-down significantly decreased TGF-β-induced VISTA expression in Jurkat T cells [39].

VISTA is a highly conserved immune checkpoint protein across the species, with a distinctive cysteine pattern which is retained from zebrafish to human [35]. Studies have reported a 76% sequence identity in VISTA gene between the human and mice, with a 90.6% identity in the cytoplasmic tail [34]. Despite showing sequence homology with the IgV domains of the CD28 and B7 families, it is significantly different from B7 family members both functionally and structurally [35]. Four invariant cysteines (one in the stalk and three in the Ig-V domain) as the unique structural characteristics of VISTA shape its novel tertiary and/or quaternary structure and lead to unique disulfide bonding patterns [35]. In contrast to the other members of the B7 family, VISTA has 10 beta strands, an extra helix which constitutes a specific epitope for VISTA interactions, a C-C′ loop (including seven charged, surface-exposed residues), and two extra disulfide bonds, however it lacks an immunoglobulin constant (IgC) domain [37]. VISTA has three C-terminal SH3-binding domains and one conserved Src homology 2 (SH2)-binding motif in the center, in absence of immunoreceptor tyrosine-based inhibitory motif (ITIM)/ immunoreceptor tyrosine-based activation motif (ITAM) motifs in the cytoplasmic tail [40].

VISTA is proposed to function both as a receptor and a ligand. The extracellular IgV-like domain enable ligand activity by binding to PSGL-1 or VSIG-3, while its cytoplasmic tail, containing SH2- and PKC-binding motifs and proline-rich docking sites, mediates receptor-type inhibitory signaling. Interestingly, VISTA lacks an ITIM-like motif and has demonstrated sequence homology to both PD-L1 and PD-1; with its IgV domain sharing about 23% identity with PD-L1. VISTA possesses a unique cytoplasmic tail with SH2-, SH3-, and PKC-binding motifs, indicating a distinct yet functionally analogous inhibitory mechanism. However, PD-1 contains ITIM and ITSM motifs that recruit phosphatases for inhibitory signaling [17, 34]. Numerous casein kinase 2 and phosphokinase C phosphorylation sites in the cytoplasmic tail could be used to initiate signaling [37]. VISTA expression on T cells as a receptor transmits inhibitory signals. However, its expression on APCs as a ligand and its binding to an unidentified receptor on T cells inhibit downstream signaling. Studies using a VISTA Ig fusion protein (VISTA-Ig) demonstrated reduced CD8^+^ and CD4^+^ T cells proliferation and cytokine production including, IFN-γ, IL-2, and TNFα. Suppressive effects of VISTA are mainly through decrease of inhibiting T-cell proliferation and activation, which can be accompanied by modulation of markers including CD69, CD44, and CD62L, although it has a minimal direct impact on apoptosis [21, 35, 36]. Using an agonist mAb against PD-1 H as well as PD-1 H knock-out mice demonstrated the increase of anti-tumor activation, suggesting that PD-1 H suppresses antigen-specific TCR dependent responses by T cells [21].

## VISTA ligands

### VSIG3

V-Set and Immunoglobulin domain containing 3 (VSIG3), also referred as immunoglobulin superfamily 11 gene (IgSF11), CT119, Igsf13, CXADRL1, brain- and testis-specific Ig superfamily protein (BT-IgSF), and BTIGSF is a type I transmembrane protein that belongs to the coxsackie virus and adenovirus receptor (CAR) subgroup of the adhesion molecules. The gene responsible for encoding the protein is found on chromosome 3q13.32; the protein has 431 amino acids and is widely expressed in testis and brain [41, 42]. Having a membrane-distal V-type domain and a membrane-proximal C2-type domain in the ECD (219 aa), the protein has a high homology with CAR, CAR-like membrane protein (CLMP), and endothelial cell-selective adhesion molecule (ESAM) [43]. IgSF11 also contains a transmembrane domain (21 aa) and a PDZ-binding motif at C-terminus (cytoplasmic domain with 169 aa) which mediate the interactions with intracellular proteins [44]. IgSF11 mediates not only neural development but also the synapse formation. Previous studies suggest a tripartite complex formation among IgSF11, postsynaptic scaffold protein PSD-95 and α-amino-3-hydroxy-5-methyl-4-isoxazolepropionic acid receptor (AMPARs) which regulates AMPAR-mediated synaptic transmission and plasticity [44]. IgSF11 is also expressed in the blood–testis barrier (BTB) and the homozygous BT-IgSF knock-out mice demonstrated a reduction in the testis size, azoospermia, and male infertility, suggesting that BT-IgSF is probably required for a functional BTB [45]. VSIG3 is highly expressed in hepatocellular carcinomas, colorectal cancers, and intestinal-type gastric cancers, suggesting its potential role for cancer immunotherapy [46]. Studies reported that three isolated VISTA residues (R54, Q63, and F62) are probably involved in VISTA-VSIG3 binding interactions [37]. The interaction of VISTA and VSIG3 moderately depends on pH and is effectively enhanced in neutral conditions. While the binding affinity is about 20nM in pH 7.4, it is around 80nM in pH 6.0 as acidic environment such as TME [42, 47]. This interaction may inhibit the proliferation of human T cells as well as the production of cytokines and chemokines such as IL-17, IL-2, IFN-γ, chemokine (C-C motif) ligand 3 (CCL3), (C-C motif) ligand 5 (CCL5), and C-X-C motif chemokine 11 (CXCL11) in vitro, suggesting that VSIG3 can negatively regulate the immune responses [48].

### Galectin-9

Galectin-9 also known as HUAT and LGALS9A is a member of the beta-galactoside-binding proteins encoded by LGALS9 gene on chromosome 17q11.2. Galectin-9 contains two carbohydrate recognition domains (CRDs) joined by a peptide linker. Three isoforms of galectin-9 could be present in different individuals based on the length of the polypeptide linker [35, 37]. Galectin-9 facilitates immune escape of tumor cells through interaction with T cell immunoglobulin and mucin-containing protein 3 (Tim-3) and decreasing NK cell cytotoxicity [49]. Specific interactions between galectin-9 containing media and VISTA, using ELISA method with an anti-VISTA antibody coated plate suggested that galectin-9 could be considered as another VISTA ligand and further experiments reported a high binding affinity. Galectin-9 and soluble VISTA interactions can also induce apoptosis due to an increase in the granzyme B activity within the cytotoxic T cells [50]. Recent findings demonstrated that T cell-induced secretion of galectin-9 from various human cancer cells derived from solid malignant tumors, including breast and kidney cancer and high grade glioblastoma cells [51].

### PSGL-1

P-selectin glycoprotein ligand-1 (PSGL-1) adhesion molecule also known as CD162 is a trans membrane protein encoded by selectin P ligand (SELPLG) gene on chromosome 12q24.1, carrying out the cutaneous lymphocyte–associated antigen (CLA) as a glycosylation-dependent epitope. It is considered as a receptor for P-, E- and L- selectins and is commonly expressed on lymphoid and myeloid cells, and platelets [52]. The protein contains an extracellular domain which needs sulfated-tyrosine residues at N-terminus site while binding to P- and L-selectin and the highly conserved transmembrane and cytoplasmic domains [53, 54]. The binding of PSGL-1 on T cells to endothelial cells promotes migration of leukocytes to the inflammatory sites [55], however, it can negatively regulate T cell immune responses and restrain anti-tumor responses by inducing T cell exhaustion [56]. The interaction of VISTA and PSGL-1 is selectively enhanced at acidic pH. This interaction is probably through protonated histidine residues (including H153, H154 and H155) and negatively charged sulfated tyrosine as well as glutamic acid residues [22]. TCR signaling inhibition and immune suppression are caused by the interaction of VISTA and PSGL-1 on T cells [57].

## VISTA expression profile on immune cells and role in TME

**Fig. 2:**
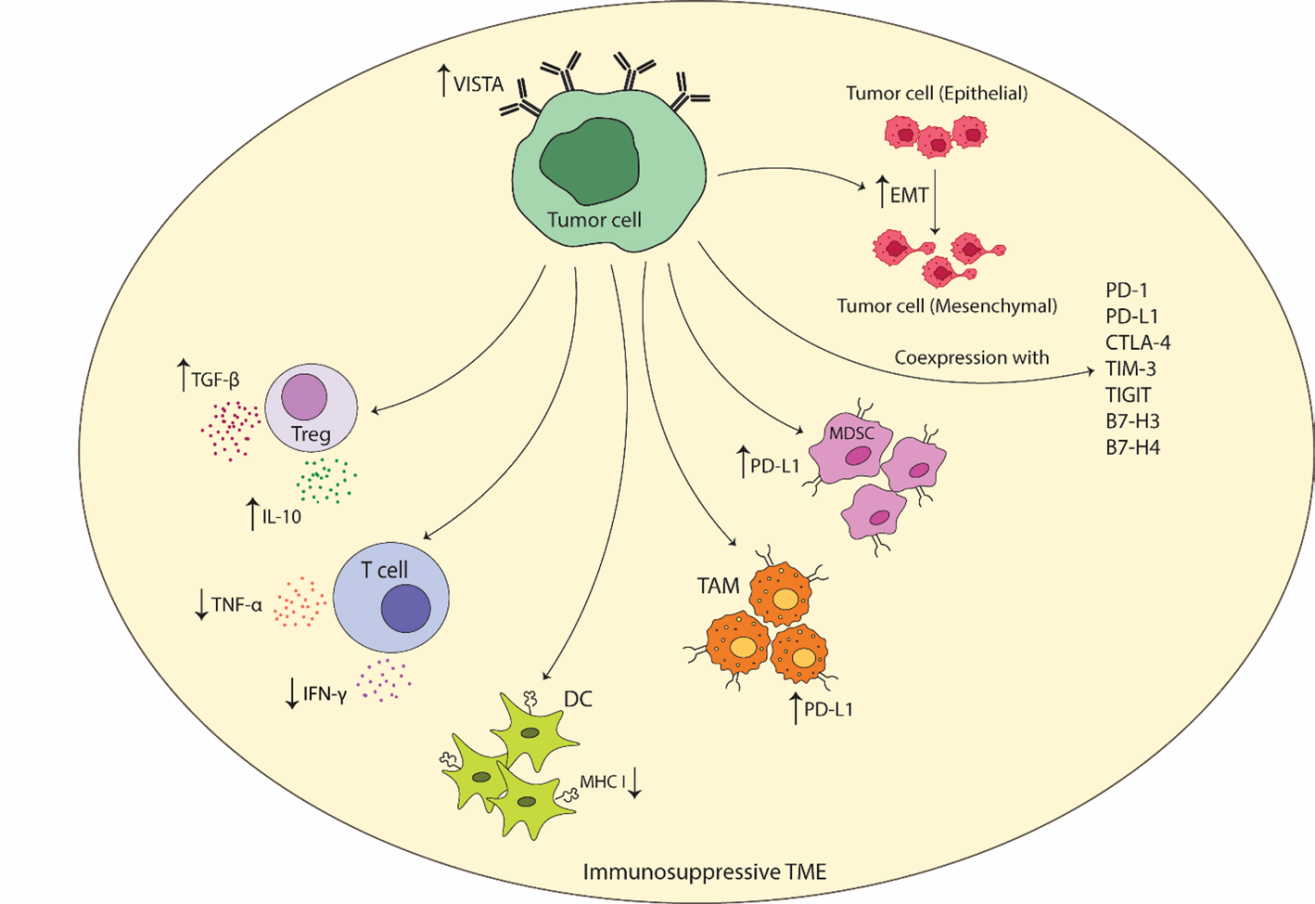
Role of VISTA in tumor immunosuppressive microenvironment. VISTA: V-domain Ig suppressor of T cell activation. PD-L1: Programmed death-ligand1. CTLA-4: Cytotoxic T-lymphocyte associated protein 4. TIM-3: T cell immunoglobulin and mucin-domain containing-3. TIGIT: T cell immunoglobulin and ITIM domain

VISTA plays a critical role in shaping the TME by modulating immune cell activity and maintaining an immunosuppressive milieu. It has been reported that VISTA expression contributes to the establishment of an immunosuppressive TME (Fig. 2). VISTA is discovered to be one of the prevalent immune checkpoint receptor within the TME of various cancer types such as melanoma, glioma, and breast cancer [58–60]. The investigation of epithelial and mesenchymal markers in the TME has demonstrated a negative association between VISTA expression and epithelial markers, as well as a positive association with mesenchymal markers. This suggests that VISTA may play a role in tumor metastasis in breast cancer [61]. In comparison to CTLA-4, PD-1, PD-L1, lymphocyte activation gene-3 (LAG-3), T cell immunoreceptor with Ig and ITIM domains (TIGIT), and TIM3, VISTA expression has been found to be the highest on immune cells in breast cancer [60]. VISTA has displayed the highest expression in glioma among CTLA-4, PD-1, LAG-3, TIGIT, and TIM3 [59]. Elevated levels of inhibitory cytokines, including IL-10 and TGF-β, are found to be associated with high VISTA expression in glioma tumors [59]. In extranodal natural killer/T-cell lymphoma (ENKTCL), VISTA expression is reported to be more prevalent than PD-L1 [62]. As shown in Table 1, several types of cells including tumor cells, immune cells, and endothelial cells express VISTA in TME. In human normal tissues, VISTA is predominantly observed in hematopoietic tissues or tissues with a substantial population of infiltrating leukocytes [36]. Interestingly, CD45^+^ (the leukocyte common antigen) cells have been the main VISTA-expressing cells in TME of renal cell carcinoma and colorectal carcinoma [63, 64]. The expression of VISTA is reported to be higher in the immune cells than in the tumor cells in triple-negative breast cancer (TNBC) [65]. In pancreatic ductal adenocarcinoma (PDAC), VISTA-expressing cells in TME were often identified as non-tumor cells rather than tumor cells [66]. In PDAC, VISTA has repressed the production of IFN-γ and TNF-α from CD4^+^ and CD8^+^ T cells [66]. VISTA^+^ tumors have displayed an increase in the frequency of regulatory T cells, a reduction in the expression of major histocompatibility complex (MHC) class II on DCs, and an upregulation in PD-L1 expression on TAMs and MDSCs in melanoma [67]. The expression of VISTA on tumor-infiltrating Tregs elevated when compared to those from peripheral lymph nodes. This indicates that VISTA expression on Tregs might influence the suppression of tumor-specific immunity in TME. VISTA was reported to regulate the suppressive function of Foxp3^+^CD4^+^ natural Tregs, as well as the *de novo* induction of induced Tregs [68]. VISTA expression was also higher on CD62L^−^ and ICOS^−^ Treg subsets, in comparison to CD62L^+^ and ICOS^+^ Treg subsets [68]. Moreover, VISTA expression has exhibited a positive correlation with the expression of other types of immune checkpoint molecules such as PD-1, PD-L1, PD-L2, TIM-3, TIGIT, CTLA-4, B7-H4, and B7-H3 [60–62, 69–77].

Myeloid cells, particularly CD68^+^ TAMs, are reported to be the major VISTA-expressing immune cells in TME [58, 60, 62, 75, 76, 78, 79]. It is highly expressed on monocytes and macrophages, as a potent negative regulator of T cell function [76]. A significantly higher expression of VISTA was detected on CD68^+^ macrophages in pancreatic cancer as an immune checkpoint therapy-resistant tumor, highlighting its role as a relevant target for effective treatment [15]. Moreover, in esophageal adenocarcinoma and breast cancer, higher expression of VISTA was reported on tumor-infiltrating CD68^+^ macrophages than T cells [29, 60], which reversed in NSCLC [77]. VISTA is constitutively released from human monocytes in peripheral blood, which is regulated via matrix metalloproteinases. However, macrophage-induced cytokines such as granulocyte–macrophage colony-stimulating factor (GM-CSF) and macrophage colony-stimulating factor (M-CSF) led to a reduction of VISTA release. Interestingly, M1 macrophages produced more VISTA than M2 macrophages, and a reduction in the release of soluble VISTA was observed following stimulation of activated macrophages with toll-like receptor 4 (TLR4). Although soluble VISTA inhibited T cell cytotoxicity, it didn’t cause programmed cell death, implying that VISTA is continuously released in peripheral circulation, possibly leading to peripheral tolerance [80]. In clear cell renal cell carcinoma (ccRCC), VISTA^+^ TAMs are discovered to be significantly higher in the TME than in peripheral blood mononuclear cells (PBMCs) and para-tumor tissues. However, the VISTA expression on myeloid dendritic cells (mDCs) or MDSCs has been similar among PBMC, para-tumor, and tumor tissues [63]. Additionally, other myeloid cells like DCs, monocyte, and tumor-infiltrated neutrophils have expressed VISTA in TME [58, 76]. Besides TAMs, monocytic and granulocytic MDSCs (mMDSCs and gMDSCs), mDCs and plasmacytoid DCs (pDCs), express VISTA in TME of colorectal cancer (CRC) [64]. However, VISTA expression was mostly observed in mMDSCs and monocytes in the periphery of CRC patients. In addition, VISTA expression on all subsets was significantly higher in the TME than in the periphery [64]. Furthermore, CD33 expression has demonstrated a significant positive correlation with VISTA expression in melanoma [79].

VISTA is reported to highly expressed on tumor-infiltrating MDSCs, known as a heterogeneous population of immature cells that accumulated in TME to suppress immune responses [81]. Regions with profound hypoxia in TME revealed higher expression of VISTA on MDSCs which was associated with worse overall survival in colorectal cancer patients [82]. In TME of melanoma mouse models, VISTA was highly expressed by MDSCs and Foxp3^+^CD4^+^ Tregs but not tumor cells. VISTA blockade resulted in a decrease in MDSC percentage, an impairment of Foxp3^+^CD4^+^ regulatory T cells suppressive function, and an increase in the presence of activated DCs [83]. In addition, VISTA is highly expressed on MDSCs in AML patients compared to healthy controls, mediating the inhibition of T cell responses [16]. Collectively, high expression of VISTA on MDSCs is indicative of poor prognosis and immune suppression, which needs to get more attention as an immunotherapeutic target.

In addition to myeloid cells, lymphocytes including, CD3^+^ T cells, CD4^+^ T helper cells, CD8^+^ cytotoxic T cells, and CD19^+^ B cells are demonstrated to express VISTA in TME [58, 75, 77].

Little is known about the VISTA expression by B cells. Single-cell RNA-seq analysis revealed low expression of VISTA by B cells in comparison to CD68^+^ TAMs, CD4^+^ T cells and CD8^+^ cytotoxic T cells in breast cancer [60]. Furthermore, VISTA level was positively associated with CD68^+^ macrophages, CD3^+^ T cells, and CD19^+^ B cells in pancreatic cancer. A higher expression level was also detected on tumor-infiltrating CD68^+^ macrophages in comparison to T and B cells [75]. However, VISTA expression has not been detected in CD19^+^ B cells in breast cancer, in CD3^+^ T cells in CRC, as well as in CD8^+^ T cells in esophageal adenocarcinoma (EAC) [29, 60, 78].

VISTA regulates T cell quiescence and peripheral tolerance, in addition to limiting T cell activation and inducing states of distinct dysfunction [36, 84]. The expression of VISTA by T cells is confirmed by different studies on human and mouse models. VISTA is elevated within the TME, in which the blockade is associated with enhanced immune responses in mice. The VISTA expression on CD4^+^ and CD8^+^ T cells increased after stimulation and particularly after co-culture with tumor cells, besides an elevation in expression level of pro-inflammatory cytokines, which was reversed following the addition of recombinant VISTA. Moreover, VISTA blockade on T cells caused the reduction of tumor weights in vivo [66]. High VISTA expression associated with CD8^+^ tumor infiltrating cells was reported in hepatocellular carcinoma. Additionally, the dual positive expression of VISTA^+^/CD8^+^ revealed favorable TME and better overall survival which was seen in ovarian cancer as well [31, 85]. In breast cancer TME, CD4^+^ and CD8^+^ T cells expressed VISTA, while it was higher on CD68^+^ TAMs [60]. However, VISTA expression was shown to be higher in T cells in comparison to macrophages; and in cytotoxic T cells than in T helper cells in human NSCLC [77]. The expression of VISTA on CD4^+^ T cells in NSCLC patients has been associated with suppressing the production of cytokines related to Th1 (IFN-γ, IL-2), Th2 (IL-4), Treg (IL-10), Th17 (IL-17), and macrophage (IL-12P70) [86]. In esophageal adenocarcinoma, a positive co-expression of VISTA with CD68^+^ and CD4^+^ cells was seen, while no reliable co-expression with CD8^+^ cells was detected [29]. In TNBC and PDAC, VISTA expression has displayed a positive correlation with the infiltration of CD3^+^ T cells, CD4^+^ T cells, CD8^+^ T cells, CD19^+^ B cells, as well as CD68^+^ TAMs [65, 75, 87]. The same observation has been reported in NSCLC, except for the CD4 expression that was negatively correlated with the VISTA expression [77]. In ovarian cancer, VISTA expression in the immune cells is found to be significantly associated with higher infiltration of CD3^+^, CD4^+^, Foxp3^+^, and CD8^+^ lymphocytes [31]. In ENKTCL, VISTA expression has been positively correlated with the infiltration of CD8^+^ and Foxp3^+^ tumor infiltrating lymphocytes (TILs) [62]. In a more detailed analysis, Zhang et al., indicated that in TNBC patients with high VISTA expression, tumor infiltrated cells are consisted of memory B cells, CD8^+^ T cells, resting and activated CD4^+^ memory T cells, regulatory T cells, monocytes and M1 macrophages, while in low VISTA-expressing tumors, naïve B cells, T gamma delta cells, T follicular helper cells, resting NK cells, M0 macrophages as well as resting and activated mast cells are the prominent infiltrated immune cells [87]. In gastric carcinoma and gastroesophageal junction cancer (GEJ), VISTA has been detected not only in primary tumors but also in corresponding liver metastases, in both tumor and immune cells. However, the expression of VISTA in primary tumors couldn’t predict its expression in metastases and vice versa [70].

There are pieces of evidence suggesting the involvement of VISTA in mechanisms associated with resistance to cancer treatment. To gain insight into immunotherapy resistance, the overexpression of TIM3/VISTA and CD68 was demonstrated to be significantly associated with shorter progression-free survival after anti-PD-1/PD-L1-based therapies [88]. In melanoma, the frequency of VISTA^+^ lymphocytes in tumor tissues is shown to be upregulated in patients who developed acquired resistance to treatment with immune checkpoint inhibitors [89]. In prostate cancer, VISTA expression on CD4^+^ T cells, CD8^+^ T cells and CD68^+^ macrophages increased after ipilimumab therapy [90]. In addition, neoadjuvant therapy resulted in the upregulation of VISTA in patients with gastric carcinoma, which was also associated with resistance to therapy [91]. Collectively, VISTA exhibits wide expression within the TME and its expression is associated with the infiltration of various immune cells, the formation of an immunosuppressive TME, the expression of multiple other immune checkpoint molecules, and resistance to treatment.

## Clinical significance and prognosis

VISTA expression in tumors has been increasingly recognized due to its potential impact on clinical outcomes and patient prognosis. The association of VISTA with clinical and pathological features, as well as its prognostic role, varies significantly across different kinds of cancers. Kuklinski et al. reported that VISTA could serve as an independent negative prognostic marker in primary cutaneous melanoma. Additionally, their findings demonstrated a positive correlation between VISTA expression and neutrophils infiltration, which, in turn, was associated with ulceration [76]. Moreover, VISTA expression was shown to be associated with unfavorable clinicopathological features such as lymph node involvement, presence of ulceration, deeper Breslow thickness, higher frequency of vertical growth phase, and advanced stage in melanoma [79].

In breast cancer, in both protein and gene expression analysis, VISTA has been linked to unfavorable clinicopathological factors [60, 61]. In addition, exploring the expression of VISTA on immune cells and tumor cells separately, revealed a link to undesirable clinicopathological features in breast cancer [69]. However, in patients with estrogen receptor (ER)-negative, progesterone receptor (PR)-negative, and basal-like invasive ductal carcinoma (IDC), the expression of VISTA on immune cells was correlated with a positive prognosis and served as an independent indicator for improved relapse-free survival (RFS) and disease-specific survival (DSS) [69]. Furthermore, in TNBC, survival analysis has revealed that VISTA is a positive prognostic factor [65, 87].

A higher infiltrated CD4^+^ VISTA^+^ T cells/ CD4^+^ VISTA^−^ T cells ratio was associated with a higher pathological node (pN) stage in NSCLC patients [86]. Ma et al. showed that higher expression of VISTA is associated with shorter overall survival (OS); however, Villarroel-Espindola et al. revealed an association between VISTA expression in tumor tissues and a longer 5-year OS in NSCLC [77]. In ccRCC with venous tumor thrombus, the expression of VISTA in tumor thrombus was positively associated with metastasis, tumor necrosis, and reduced OS, while VISTA expression in primary tumors was associated with smaller tumor size [92]. In CRC, enhancement of VISTA mRNA expression in tumor tissues of advanced stages in comparison to the early stages has been detected, although it was not significant [93]. However, a favorable clinicopathological feature and longer survival is reported in CRC patients with high expression of VISTA [78, 94].

In gastric carcinoma and GEJ, the expression of VISTA was found to be associated with Laurén classification as well as tumor localization and to exhibit variations during tumor progression [70]. VISTA was reported to be increased after platinum-based neoadjuvant treatment in patients with gastric carcinoma and GEJ, revealing higher expression in patients with minimal or no response to therapy [91]. VISTA expression did not hold prognostic value for gastric carcinoma and GEJ patients, regardless of whether they received neoadjuvant treatment or not [70, 91]. In EAC, the high frequency of VISTA^+^ TILs was associated with favorable clinicopathological features including early (pT1/2) tumor stages, negative lymph node involvement, and early UICC stages (stage I/II). Moreover, VISTA served as a prognostic factor for improved OS in EAC, especially in the early tumor, node, metastasis (TNM) stages [29].

In ovarian cancer, high VISTA expression in immune and tumor cells is reported to be associated with aggressive pathological features (advanced tumor stage and lymph node metastasis) [95]. Regarding the prognostic value of VISTA in ovarian cancer, no associations were found between VISTA expression (neither in immune cells nor in tumor cells) and OS in two investigations [31, 95]. However, Zong et al. have shown that VISTA expression in tumor cells is associated with prolonged progression-free survival (PFS) and OS in high-grade serous ovarian cancer [71]. Combinatorial analysis of VISTA and CD8 expression in the TME of ovarian cancer revealed that dual expression of CD8 and VISTA is correlated with the best OS, whereas, VISTA expression alone is correlated with the shortest OS [31].

In endometrial cancer, the expression of VISTA in immune cells exhibited significant elevation during the early stages and has been suggested as a predictor of improved survival [74]. In cervical cancer, VISTA on immune cells was predominantly detected in the primary than in recurrent tumors and in squamous cell carcinoma in comparison to adenocarcinomas [64]. VISTA was higher in moderate- and poor-differentiated carcinomas as well. Moreover, VISTA positivity on immune cells, as well as double positivity of VISTA and B7-H4, was associated with improved survival [72]. However, in HPV-infected cervical cancer, VISTA was found to be an independent prognostic factor for shorter PFS [96]. In PDAC, VISTA was predominantly detected in non-survivor patients and its expression was significantly correlated with shorter OS [97]. The expression of VISTA in tumor cells was associated with longer OS in PDAC [75]. Simultaneous analysis of PD-L1 and VISTA showed that the expression of both VISTA and PD-L1 is associated with the lowest OS, whereas, the absence of both markers was associated with the highest OS in PDAC [97]. In pancreatic neuroendocrine tumor (panNETs), elevated VISTA^+^ microvessel density (MVD) was linked to undesirable pathological characteristics and was identified as an independent prognostic factor of shorter PFS in patients [98].

An earlier investigation on oral squamous cell carcinoma (OSCC) revealed a significant correlation between VISTA expression and lymph node status [99]. However, Starzynska et al. failed to obtain similar results [100]. VISTA did not show prognostic value for OSCC, however, exploring the expression of VISTA and CD8 simultaneously revealed that patients with high VISTA and low CD8 expression had shorter survival than other patients [99]. Moreover, OSCC patients with higher expression of VISTA exhibited a higher 5-year disease-free survival (DFS) rate [100]. In hepatocellular carcinoma (HCC), VISTA was predominantly detected in patients with higher pathological grades (III-IV) and without liver cirrhosis [85]. Shrestha et al. reported high expression of VISTA in high-risk HCC patients [101]. Analysis of VISTA and CD8 concurrently showed that the dual expression of CD8 and VISTA was associated with the longest OS [85]. VISTA in tumor cells was negatively associated with liver cirrhosis and tumor size. Moreover, patients with VISTA positivity in tumor cells, but not immune cells, displayed significantly longer OS [85]. However, Shrestha et al. revealed that high VISTA expression, alone or in combination with PD-L1 expression, was significantly associated with lower OS and RFS in high-risk HCC patients [101].

In soft tissue sarcoma, VISTA was more frequently detected in patients with higher tumor grade (G3 vs. G2), however, VISTA^+^ tumors were associated with longer survival in STS patients [102]. In ENKTCL, high VISTA expression was significantly detected in patients with unfavorable clinical outcomes. Exploring the expression of VISTA in combination with PD-L1 demonstrated that high expression of both VISTA and PD-L1 was associated with the shortest PFS and OS in ENKTCL [62]. In glioma, the expression of VISTA was significantly upregulated in grade III/IV compared to grade I/II. Moreover, patients with lower VISTA expression exhibited prolonged survival [59].

In bladder cancer, VISTA expression in immune cells was significantly correlated with higher tumor stage, higher pathologic grade, larger tumor size (> 3 cm), and multiple bladder cancer lesions. Patients with VISTA^+^ immune cells represented poorer intravesical RFS than VISTA^−^ tumors [73]. In conclusion, findings on the prognostic role of VISTA are highly controversial and strongly depend on the tumor type and the evaluation techniques. Nevertheless, in a systematic evaluation, He et al. determined that high VISTA expression is associated with improved OS, probably due to the high infiltration of CD8^+^ cells [103].

**Table 1:** Expression, clinicopathological significance, and prognostic value of VISTA in different cancers

Cancer type	Used technique	VISTA-expressing cells	VISTA expression difference between tumoral and normal tissues	Association of VISTA expression and clinicopathological characteristics of patients	Prognostic value of VISTA expression	Ref
Cutaneous melanoma	IHC	Tumor-infiltrated neutrophils and mononuclear cells	Not studied	Not studied	Independent negative prognostic factor	[76]
Cutaneous melanoma	IHC and IF	CD33^+^ MDSCs	Not studied	Positive correlation with lymph node involvement, presence of ulceration, deeper Breslow thickness, higher frequency of vertical growth phase, and advanced stage	Association with worse survival	[79]
Breast cancer (Invasive ductal carcinoma)	IHC	ICs and TCs	Not studied	**On ICs**: more frequent in ER-negative, PR-negative, and HER2-positive patients and associated with younger age, poor differentiation, and triple-negative status **On TCs**: more frequent in ER-negative, PR-negative, HER2-positive, triple-negative, and unfavorable molecular subtypes	Independent prognostic factor in terms of improved RFS and DSS in patients with ER-negative, PR-negative, and basal-like subtypes	[69]
Breast cancer	IHC and single cell RNA-seq	ICs and TCs	Higher levels in tumor tissues than adjacent normal tissues (RNA-seq)	Positive correlation with pathological grade and lymph node status	No association with OS	[60]
Breast cancer	TCGA and TISCH data (RNA-seq) analysis, RT-PCR, and IHC	ICs and TCs	Higher levels in tumor tissues than adjacent normal tissues (IHC)	Positive association with lobular and metaplastic histological subtypes, larger tumor size, lymph node status, and negative ER status. (TCGA data) lower in Luminal B than Luminal A and HER2 + molecular subtypes (RT-PCR) **On ICS**: upregulation in TNBC, positive association with tumor grade, ER-negative status, and PR-negative status (IHC) **On TCs**: upregulation in TNBC and patients with larger tumor sizes (IHC)	Not studied	[61]
Triple-negative breast cancer	TCGA and FUSCC data (RNA-seq) analysis and IHC	TCs and stromal cells	Lower levels in tumor tissues than in adjacent normal tissues (TCGA data)	Higher levels in patients aged ≤ 60 than in patients aged over 60 (FUSCC data)	High mRNA levels were associated with better RFS and OS	[87]
Triple-negative breast cancer	TCGA data (RNA-seq) analysis and IHC	ICs and TCs (Exclusively cytoplasmic)	Not studied	**On ICs**: correlated with no lymph node metastasis, AJCC stage I and II, and basal-like subtype **On TCs**: no correlation between VISTA on TCs and the clinicopathological characteristics of patients	Positivity on ICs was the only prognostic factor for both RFS and OS in T1-2N0 patients	[65]
Lung adenocarcinoma	IHC and multiplex IF	ICs and TCs	In cancer tissues was significantly higher than noncancerous tissues	VISTA expression in IC was correlated with smoking history	High VISTA expression in IC predicted poorer survival	[104]
Non-small-cell lung cancer	IF	ICs, TCs, and stromal cells	Higher levels of stromal VISTA in tumor tissues compared to normal lung tissues from the same cases	No consistent association between VISTA levels and clinicopathological characteristics of patients	Expression in the tumor area was associated with longer OS	[77]
Non-small-cell lung cancer	IHC and multicolor IF	CD45^+^, CD4^+^, and CD8^+^cells	Higher frequency of CD4^+^VISTA^+^ T cells in tumor tissues in comparison to pre-tumor tissues	Higher infiltrated CD4^+^ VISTA^+^ T cells/ CD4^+^ VISTA^−^ T cells ratio was associated with a higher pathological node (pN) stage	VISTA expression on CD4^+^ T cells was correlated with the shorter OS	[86]
Clear cell renal cell carcinoma	IF and TCGA data (mRNA expression level) analysis	TCs and ICs	Higher in tumor tissues compared to non-tumoral tissues (TCGA data)	Not studied	Not studied	[63]
Clear cell renal cell carcinoma	TCGA data (RNA-seq) analysis and IHC	Macrophage	Higher in tumor tissues compared to normal renal parenchyma (TCGA data and IHC)	Not studied	Not studied	[105]
Renal cell carcinoma with venous tumor thrombus	IHC	ICs but not TCs	Not studied	Positive association with gender, the presence of distant metastases, and tumor necrosis in the venous thrombus Positive association with smaller tumor size in primary tumors	No prognostic value in primary tumors Association with poor OS in venous tumor thrombi	[92]
Colorectal cancer	RNA-seq data analysis, RT-PCR, and IF	ICs and TCs	Lower VISTA levels in tumor tissues than in normal tissues (RNA-seq data analysis and RT-PCR) Lower VISTA levels in para-tumor and normal tissues in comparison to tumor tissues (IF)	Not studied	No association was found (RNA-seq data analysis)	[64]
Colorectal cancer	TCGA data (RNA-seq and whole exome seq) analysis	Not studied	Lower in tumor tissues compared to normal tissues	Not studied	No difference between the low and high expression of VISTA in terms of survival	[106]
Colorectal cancer	RT-PCR	Not studied	Relatively similar mRNA levels in tumor tissues and normal tissues Higher mRNA levels in the circulation of patients, compared to healthy individuals	Higher in advanced stages than early stages (non-significance) A trend towards higher expression in patients with high grades of tumor budding	Not studied	[93]
Colorectal cancer	IHC, IF	ICs and TCs	Not studied	High VISTA expression on ICs was more frequent in patients with N0 stage, T1-2 stage, and low tumor grade	Positive association with better survival and an independent positive prognostic factor	[78]
Colorectal cancer	IHC	Not studied	Not studied	Association with lower AJCC stage and mature stromal differentiation	Association with a better prognosis	[94]
Gastric carcinoma and gastroesophageal junction cancer	IHC	TCs (only cytoplasmic), ICs and ECs	Not detected in non-neoplastic gastric epithelium	Association with Laurén phenotype (intestinal and unclassified > diffuse) and tumor localization (proximal > distal stomach)	No association was found	[70]
Gastric carcinoma and gastroesophageal junction cancer (after platinum-based neoadjuvant chemotherapy)	IHC	TCs (only cytoplasmic), ICs and ECs	Not detected in non-neoplastic gastric epithelium	Positive association with tumor regression grade Elevation from stage I to III and reduction thereafter	No association was found	[91]
Esophageal adenocarcinoma	IHC	TCs and ICs	Not studied	Positive association with early (pT1/2) tumor stages, negative lymph node involvement, and early UICC stages (stage I/II)	Prognostic factor for improved overall survival, especially in the early TNM stages	[29]
Ovarian cancer	IHC	Tumor cells, ICs, and endothelial cells	Not studied	Positive association with advanced stage and lymph node metastasis (on ICs or TCs) Association with lymph node metastasis (on ECs)	No association was found	[95]
Ovarian cancer	IHC	TC and IC	Normal ovarian tissue (negative control)	Higher expression in higher stages, distant metastasis, and high-grade serous carcinoma subtype (non-significant).	No association was found	[31]
Ovarian cancer	IHC and TCGA data (mRNA expression level) analysis	TCs, ICs, and endothelial cells	Not studied	Lower on both the TCs and ICs of mucinous carcinomas than those of serous carcinomas Lower in the ICs of clear cell carcinomas than serous carcinomas	Association with favorable prognosis in patients with high-grade serous ovarian cancer	[71]
Endometrial cancer	IHC	TCs and ICs	Not studied	**On ICs**: more frequent in early-stage disease **On TCs**: more frequent in patients ≥ 58 years of age and clear cell carcinoma histological type	Predictor of improved survival	[74]
Cervical cancer	IHC	ICs and TCs	Not studied	**On ICs**: more frequent in primary tumors than in recurrent counterparts and in moderate- and poor-differentiated carcinomas **On ICs and TCs**: more frequent in squamous cell carcinoma than in adenocarcinomas	Independent predictor of favorable outcomes	[72]
HPV-infected cervical cancer	IHC	ICs and TCs	Not studied	No association was found	Shorter survival in patients with VISTA positivity An independent prognostic factor for PFS	[96]
Pancreatic ductal adenocarcinoma	IHC, multiplex IF, TCGA data (RNA-seq) analysis, and multicolor flow cytometry	TCs and ICs	Higher in tumor tissues than normal tissues (IHC and RNA-seq data analysis)	No association was found	A trend towards worse overall survival in patients with high VISTA expression on TCs	[66]
Pancreatic ductal adenocarcinoma	IHC, multiplex IF and TCGA data (RNA-seq) analysis	ICs, TCs, and ECs	Barely expressed in the adjacent normal tissues	Not studied	VISTA expression in TCs is an independent prognostic factor for improved OS	[75]
Pancreatic ductal adenocarcinoma	IHC	TCs	Not studied	Not studied	VISTA expression in the center only and in both the center and the periphery of tumors, but not in the periphery only, was correlated with shorter OS Dual expression of VISTA and PD-L1 was associated with the lowest OS	[97]
Pancreatic neuroendocrine tumors	IHC	ICs and ECs but not TCs	Not studied	High VISTA^+^ MVD was more frequent in tumors with high tumor grade, presence of lymphovascular invasion and/or perineural invasion, high mitotic count, and high Ki-67 index VISTA on ICs was not associated with clinicopathologic parameters	High VISTA^+^ MVD was an independent predictor of shorter PFS	[98]
Oral squamous cell carcinoma	IHC	Not studied	Higher in primary oral squamous cell carcinoma than in epithelial dysplasia and normal mucosa	Correlation with lymph node status (N0 vs. N1 + N2)	VISTA expression was not an independent predictor for poor prognosis High VISTA in combination with low CD8 expression was associated with poorer OS	[99]
Oral squamous cell carcinoma	IHC and RT-PCR	Not studied	Not studied	No association was found	VISTA expression did not serve as an independent predictor for prognosis higher VISTA H-score indicates a higher 5-year DFS rate	[100]
Hepatocellular carcinoma	IHC and TCGA data (RNA-seq) analysis	ICs and TCs	Not studied	Positive correlation with high pathological grade (III-IV) and tumors without liver cirrhosis	VISTA-positivity in TCs, but not in ICs, was associated with prolonged OS The dual expression of VISTA and CD8 was associated with longest OS	[85]
Soft tissue Sarcomas	IHC	TCs	Not studied	More frequent in higher FNCLCC grade (G3 vs. G2)	VISTA expression was independently associated with prolonged survival Better survival in patients with VISTA^+^CD3^−^ tumors compared to VISTA^+^CD3^+^ patients	[102]
Extranodal natural killer/T-cell lymphoma	IHC and IF	CD68^+^ TAM	Not studied	Positive correlation with distal lymph node metastasis, advanced Ann Arbor stage, and high nomogram-revised index and prognostic index of natural killer/T cell group	high expression of both VISTA and PD-L1 is associated with the shortest PFS and OS	[62]
Glioma	RT-PCR, IHC and TCGA data (RNA-seq) analysis	ICs, TCs, and ECs	Elevated in glioma tissues compared to PBMCs of healthy donors	Positive association with glioma grades (RT-PCR & IHC) Association with glioma grades, histological type, and molecular subtype (RNA-seq)	Patients with lower VISTA expression had prolonged survival	[59]
Bladder cancer	IHC and IF	ICs and TCs	Not studied	Correlation with higher tumor stage, higher pathologic grade, larger tumor size (> 3 cm), and multiple bladder cancer lesions	Patients with VISTA^+^ ICs represented poorer intravesical recurrence-free survival than VISTA^−^ tumors	[73]

IC: immune cell, TC: tumor cell, EC: endothelial cell, IHC: immunohistochemistry, IF: immunofluorescence, RT-PCR: real-time polymerase chain reaction, TCGA: The Cancer Genome Atlas, TISCH: Tumor Immune Single Cell Hub, FUSCC: Fudan University Shanghai Cancer Center

## VISTA as a targeted therapy

### VISTA antagonist

 VISTA has emerged as a promising immunotherapeutic target, offering new insights for cancer treatment by reversing immune suppression in tumor microenvironment. Overcoming inhibitory receptor-mediated immune suppression has been a major focus of recent cancer immunotherapeutic developments. However, the routinely administered immune checkpoint inhibitors are not successfully therapeutic, and resistance to existing immune checkpoint therapies is common, calling for the identification of novel approaches [107]. VISTA has emerged as a promising target for cancer immunotherapy. VISTA blockade is shown to alter the features of TME, resulting in an increase in tumor activating-DCs and a decrease in MDSCs. Moreover, anti-VISTA therapy impaired the suppressive functions of TME by decreasing the emergence of tumor-specific Foxp3^+^CD4^+^ regulatory T cells. Applying VISTA mAb significantly reduced transplantation and inducible melanoma [83]. Testing HMBD-002 as an Fc-independent anti-VISTA antibody in human and murine models of cancer has reduced MDSC-mediated suppression of T cell activity and inhibited neutrophil migration. This treatment changed TME milieu toward a pro-inflammatory phenotype, characterized by a Th1/Th17 response [108]. Anti-VISTA antibody treatment has revealed a significant reduction in the number of metastatic nodules in the metastatic livers of mouse models of pancreatic ductal adenocarcinoma [75]. Enhancement of T cell-mediated cytotoxicity to acute myelogenous leukemia (AML) cells is demonstrated after targeting VISTA by anti-VISTA mAb [25]. Moreover, VISTA blockade is shown to prolong the survival of tumor-bearing mice [109]. Consequently, VISTA mAb-mediated blockade impairs the tumor growth and enhances antitumor immune responses.

### Combination therapy

 VISTA can be targeted in cancer immunotherapy either as monotherapy or in combination with other therapies. Although VISTA induces CD8^+^ T cell activation by converting resting and exhausted cells into functional effector cells, it didn’t show clear tumor growth regression and didn’t inhibit recruitment of regulatory T cells to the TME. Combination blockade of VISTA and CTLA-4 elicited the ratio of both CD8^+^ and CD4^+^ T / Treg cells and increased the further tumor regression [110]. Mehta et al. have recently introduced a yeast surface display to engineer an anti-VISTA antibody, which binds to a unique epitope of VISTA in mouse, human, and cynomolgus monkey with high affinity. This anti-VISTA antibody blocked the VISTA function and inhibited the engagement with both PSGL-1 and VSIG3 proteins. As monotherapy and in combination with anti-PD1, it revealed slow tumor growth in multiple syngeneic mouse models [47]. Combinatorial administration of anti-VISTA antibody and a peptide-based cancer vaccine with TLR agonists as adjuvants synergistically impaired tumor growth [83]. Consequently, VISTA-targeted approaches, either as monotherapy or in combination with additional immune-targeted strategies for cancer immunotherapy, can suppress tumor growth and increase the activated T cells following inhibition of Tregs.

### Clinical trials

Several targeted VISTA pre-clinical and clinical trials have been conducted or are currently ongoing, involving different cancers (Table 2). These trials have evaluated the efficacy and safety of VISTA inhibition. JNJ-61610588 was a fully human IgG1ĸ mAb developed by Janssen, and the first one directed against VISTA that was examined clinically for evaluation of safety and pharmacokinetics; however, it was prematurely terminated due to cytokine release syndrome (CRS) [15, 111]. The CA-170 is another anti-VISTA mAb developed by Curis/ImmuNext that was tested in combination with PDL1/PD-L2 blockade, which has completed early-phase testing. Seventy-one patients were enrolled with advanced solid tumors and lymphomas that were unresponsiveness to current therapies and showed acceptable safety results [112, 113]. The Fc-independent IgG4 anti-VISTA mAb known as HMBD-002 is under/ recruiting in clinical trial. Hummingbird Bio, Inc as the sponsor has sought to evaluate multiple doses of HMBD-002, with or without pembrolizumab, in adult patients with solid tumors [114]. CI-8993 is also a fully human IgG1ĸ anti-VISTA mAb, which is in phase 1 of dose-escalation study to determine the maximum tolerated dose in adult patients [115, 116]. However, the efficacy outcomes of HMBD-002 and CI-8993 have not been reported. Another active clinical trial is W0180, an anti-VISTA mAb by Pierre-Fabre. It is a Phase 1 dose escalation and dose expansion study, which is aimed to evaluate the developed antibody as a monotherapy and/or in combination with pembrolizumab in adults with locally advanced or metastatic solid tumors [117]. A fully human IgG1ĸ anti-VISTA mAb named CI-8993 and sponsored by Curis, Inc is going to determine the maximum tolerated dose of CI-8993 in adult patients in phase I trial. Moreover, Kineta, Inc has planned to study the KVA12123, a fully human IgG1 anti-VISTA mAb both as a monotherapy and in combination with pembrolizumab in patients with advanced solid tumors [118]. The limitation of translating the promising preclinical efficacy to the clinics seems to be related to several factors, including the variability in expression patterns across different tumor types, the complexity of the tumor microenvironment, and the compensatory upregulation of other alternative immune checkpoints. Overall, this limited clinical evidence emphasizes the need for optimization of dosing strategies, accompanied with rational combination therapies.

**Table 2:** Pre-clinical and clinical trials evaluating the efficacy and safety of VISTA Inhibition in different cancers

Drug	Type	Target	Company	Stage	Cancers	Ref
JNJ-61,610,588	IgG1 mAb	VISTA	Janssen/ImmuNext	Phase I: [D] NCT02671955	Advanced solid tumors	[111]
CI-8993	IgG1 mAb	VISTA	Curis/ImmuNext	Curis/ImmuNext Phase I: [IP] NCT04475523	Relapsed/refractory solid tumors	[115, 116]
CA-170	Small Molecule	VISTA & PD-L1	Curis/ImmuNext	Phase I: [C] NCT02812875	Advanced solid tumors or lymphomas	[112]
CA-170	Small Molecule	VISTA & PD-L1	Curis/Aurigene	Phase II: [IP] CTRI/2017/12,011,026	Advanced solid tumors or lymphomas	[113]
K01401-020 W0180 ^+/−^ Pembrolizumab	mAb	VISTA^+/−^ PD-1	Pierre Fabre	Phase Ia, 1b: [ IP] NCT04564417	Locally advance or metastatic solid tumors	[117]
HMBD-002	IgG4 mAb	VISTA	Hummingbird	Phase I/II: [IP] NCT05082610	Advanced solid tumors	[114]
KVA 12.1	KVA 12.1	VISTA	Kineta	Pre-clinical	-	[118]

VISTA: V-domain Immunoglobulin Suppressor of T cell Activation. mAb: Monoclonal antibody. IgG1 / IgG4: Immunoglobulin G subtypes 1 / 4. PD-1: Programmed Cell Death Protein 1. PD-L1: Programmed Death-Ligand 1

### PH regulation

 The TME is acidic due to the high glycolytic rate of tumor cells, deficient blood perfusion and hypoxia, which is considered as the crucial factor of tumor progression, invasion, immune suppression and resistance to therapy [119–121]. The acidity of TME affects the immune system regulation toward suppression by inhibition of the effector T cell functions and cytokine production, stimulation of regulatory T cells, reduction of the NK cells activity and etc [122]. This acidic condition is shown to allow for pH-dependent activation of VISTA based on its unique histidine rich residues on the extracellular domain [57]. Robert J. et al. declared that VISTA preferentially engages its receptor in acidic condition such as TME due to the imidazole side chain of histidine protonates at physiologically relevant pH. In addition, they hypothesized that the histidine protonation enables VISTA-targeted antibodies to distinguish the active (acidic pH) and inactive (neutral pH) states of the VISTA ligand interface, concluding that VISTA is an acidic pH-selective ligand for PSGL-1 [22]. Furthermore, at physiologic pH in the blood circulation, VISTA binds to the PSGL-1 on endothelial cells in order to roll and extravasation. However, its binding to leukocytes is hindered at this pH. Therefore, VISTA binding to its receptor is pH-dependent and acidic TME provides VISTA engagement and further suppression of the activated T cells.

## The interplay of VISTA and other immune checkpoints in tumors

VISTA interacts with other immune checkpoints to orchestrate a complex network of immunoregulation within tumors microenvironment. In numerous studies, the concurrent investigation of VISTA and PD-1/PD-L1 has unveiled a positive association in various tumor types [59, 92, 99], ]. The concurrent expression of VISTA and PD-1/PD-L1 is reported to be associated with the worst prognosis and the shortest survival [62, 125]. These findings provide evidence that VISTA and PD-1/PD-L1 might play an important synergetic or cooperative role in the pathogenesis of various tumors and collaboratively contribute to the avoidance of immune recognition. Although VISTA and PD-1/PD-L1 share many of their immune regulatory properties, VISTA’s functions are known to be non-redundant with other B7-family members [126]. More importantly, VISTA was reported to be involved in the resistance to anti-PD-1/PD-L1 therapy in different types of tumors such as ENKTCL, prostate cancer, and melanoma [62, 89, 90]. Of note, in various tumor types, VISTA has indicated higher expression compared to PD-1/PD-L1. For instance, in melanoma, VISTA expression in tumor-infiltrating cells was found in a higher proportion of samples compared to PD-L1 expression (64.4% vs. 32.9%) [127]. In breast cancer, VISTA expression was significantly higher than that of PD-1, PDL-1, and CTLA-4 [61]. Furthermore, VISTA expression was higher in patients with high-grade serous ovarian cancer compared to PD-L1 expression (51.4% vs. 8.9%) [128]. This characteristic becomes significant in tumors exhibiting co-expression of PD-1/PD-L1 and VISTA. Consequently, monotherapy with checkpoint inhibitors may yield only a restricted or even negligible impact in tumors employing a dual evasion mechanism.

Of note, in colorectal carcinoma, VISTA mRNA levels in tumor tissue were elevated in advanced disease stages, while, levels of PD-1 in both tumor tissue and circulation were reduced in advanced stages [129]. Moreover, the presence of VISTA in melanoma was associated with unfavorable pathological features and shorter disease-specific survival and was found to be a significant independent prognostic factor, however, PD-1/PD-L1 expression showed no significant association with clinicopathological variables and melanoma-specific survival [79, 127]. In RCC, PD-L1 expression did not differ significantly between tumor tissue and adjacent non-tumoral tissues, regardless of the RCC subtype. However, VISTA showed significant upregulation in ccRCC and downregulation in chromophobe RCC tumors compared to adjacent non-tumoral tissues. While the combination of anti-VISTA and anti-PD-1 treatments led to substantial tumor reduction in a murine model of ccRCC, the combined therapy failed to produce a synergistic impact on tumor growth when compared to the effects of each monotherapy [130]. These findings underscore the significance of identifying the primary immune checkpoint molecules within the TME across various tumor types and individual patients. The synergetic effect of VISTA and other immune checkpoints seems to be likely due to their complementary and non-redundant mechanisms of function. VISTA can inhibit T-cell activation at an earlier stage, potentially preventing the initial clonal expansion and effector differentiation of these cells, reducing the pool of functional effector T-cells. However, PD-1/PD-L1 leads to exhausts the remaining ones. This mechanistic interaction and function explains why monotherapy against either often fails.

Inhibiting PD-1/PD-L1 may cause suppressing exhausted T-cell population, but it does not overcome the upstream suppression by VISTA which limits the quality of the T-cell repertoire. On the other hand, blocking VISTA alone can cause initial T-cell priming but leaves them vulnerable to subsequent exhaustion via the PD-1 pathway. This is crucial for designing rational combination treatment strategies. Relying on targeting PD-1/PD-L1 alone may be less effective in certain tumors, emphasizing the need for a more refined approach to immune checkpoint therapy and the importance of other checkpoints such as VISTA.

## Role of VISTA in tumor immune checkpoint resistance

Despite of significant improvement in cancer immune checkpoint immunotherapy, only a fraction of patients respond to current treatment; however, the exact mechanism by which the resistance comes through, is not yet well known. Recently, VISTA has emerged as a key target; implicated in acquired resistance to other immune checkpoints. In a study by Hojabr Kakavand et al. a significantly increased density of VISTA + lymphocytes in metastatic melanoma patient biopsies have been observed who initially responded to anti-PD-1 alone or combination of anti-PD-1 and ipilimumab therapy but later progressed. These findings suggest an important potential role of VISTA in acquired resistance in melanoma patients treated with anti-PD-1 [131]. In addition, the overexpression of TIM-3, VISTA, and CD68 is demonstrated to be linked to shorter progression-free survival after anti-PD-1/PD-L1 therapy upon analyzing the “cancer-immunity cycle” across various cancers; indicating the considerable role of VISTA expression in anti-PD-1/PD-L1 based immunotherapy [88]. In a colorectal cancer model, monotherapy with anti-VISTA decreased the tumor growth, and combining it with anti-PD-1/CTLA-4 led to rejection of half the tumors which have had shown complete adaptive resistance in absence of anti-VISTA. In addition, anti-VISTA treatment boosted antigen-presentation pathways and decreased myeloid-mediated suppression. Anti-VISTA therapy also activated T-cell pathways distinct from those induced by anti-PD-1 therapy. While anti-CTLA-4/PD-1 therapy expanded exhausted CD8 + T cell subsets, anti-VISTA treatment increased expression of co-stimulatory genes and decreased regulators of T-cell quiescence. These findings provide crucial mechanistic insights supporting the use of anti-VISTA to overcome adaptive resistance in contemporary PD-1 and/or CTLA-4 treatments [132]. While radiotherapy has been highly successful in treating non-small cell lung cancer (NSCLC), local relapses persist and abscopal effects are rare even when combined with immune checkpoint blockers. In a study by Zhang Y etc., it is shown that inhibiting CD39 in combination with radiotherapy selectively reduces exhausted CD8 + T cells. Furthermore, combining VISTA blockade with RT synergistically reduces immunosuppressive myeloid cells. Elevated VISTA expression is linked to poor prognosis in NSCLC patients. Altogether, data offer valuable insights into acquired resistance to radiotherapy from an immune perspective and propose rational combination strategies [133].

## Conclusion

VISTA has emerged as a potent negative regulator of various tumors; orchestrating the function of immune cells regarding its high expression on MDSCs, TAMs, monocytes, and different subgroups of lymphocytes. The association of heterogeneous expression of VISTA with prognosis requires further clarification, as it shows variable correlations with long overall survival in some cancers while indicating poor prognosis in other ones. Furthermore, the VISTA expression is associated with the formation of an immunosuppressive TME. This often takes place by increasing the expression of several other immune checkpoint molecules and promoting resistance to treatment. These findings may open new insights into understanding why other immune checkpoint therapies have not achieved the expected impressive outcomes. The trends toward combinational therapy using VISTA seem to be promising. However, it is important to evaluate the optimal efficacy of combining VISTA with other immune checkpoint blockers or chemotherapeutic agents. Investigating the expression of VISTA in the periphery, TME, lymph node, and other relevant sites in different cancers could be helpful in predicting responses to VISTA blockade. Several challenges currently limit VISTA successful translation into clinical practice, including dual role as both a ligand and a receptor, along with its context-dependent function across immune and tumor cells, the heterogeneous expression among different tumor types and treatment backgrounds, which complicates the prediction of therapeutic outcomes. Future standardized detection methods, optimized dosing strategies and large-scale clinical trials will be essential to define the optimal therapeutic settings, enhance response predictability, and ensure safety in targeting VISTA for cancer immunotherapy.

## Data Availability

No datasets were generated or analysed during the current study.
